# How engagement of a diverse set of stakeholders shaped the design, implementation, and dissemination of a multicenter pragmatic trial of stroke transitional care: The COMPASS study

**DOI:** 10.1017/cts.2020.552

**Published:** 2020-11-05

**Authors:** Sabina B. Gesell, Sylvia W. Coleman, Laurie H. Mettam, Anna M. Johnson, Mysha E. Sissine, Pamela W. Duncan

**Affiliations:** 1 Wake Forest School of Medicine, Department of Social Sciences and Health Policy & Department of Implementation Science, Division of Public Health Sciences, Winston-Salem, NC, USA; 2 Wake Forest School of Medicine, Department of Neurology, Winston-Salem, NC, USA; 3 University of North Carolina at Chapel Hill, Gillings School of Global Public Health, Department of Epidemiology, Chapel Hill, NC, USA

**Keywords:** Community-based participatory research, research design, comparative effectiveness research, patient engagement, pragmatic clinical trial, stroke

## Abstract

Evidence is limited on how to synthesize and incorporate the views of stakeholders into a multisite pragmatic trial and how much academic teams change study design and protocol in response to stakeholder input. This qualitative study describes how stakeholders contributed to the design, conduct, and dissemination of findings of a multisite pragmatic clinical trial, the COMprehensive Post-Acute Stroke Services (COMPASS) Study. We engaged stakeholders as integral research partners by embedding them in study committees and community resource networks that supported local sites. Data stemmed from formal focus groups and continuous participation in working groups. Guided by Grounded Theory, we extracted themes from focus group and meeting notes. These were discussed as a team and with other stakeholder groups for feasibility. A consensus approach was used. Stakeholder input changed many aspects of the study including: the care model that treated stroke as a chronic condition after hospital discharge, training for hospital-based providers who often lacked awareness of the barriers to recovery that patients face, support for caregivers who were essential for stroke patients’ recovery, and for community-based health and social service providers whose services can support recovery yet often go underutilized. Stakeholders brought value to both pragmatic research and health service delivery. Future studies should test the impact of elements of study implementation informed by stakeholders vs those that are not.

## Introduction

A primary goal of the Patient-Centered Outcomes Research Institute (PCORI) is to advance the science of community engagement in research [1]. To date, the biomedical literature is limited and mixed on the impact of engaging stakeholders in research, but there is sufficient evidence to suggest that stakeholder engagement is indeed a promising strategy to making research findings more meaningful to patients and the providers who care for them and, as a consequence, more likely to be used and to benefit citizens [2–8].

Clinicians, social scientists, and funders have demonstrated increased interest in how to effectively involve stakeholders in all phases of research so that research is relevant, meaningful, and actionable to those receiving the intervention, delivering the intervention, and translating the model into standard practice and policy. Initially, much of the research focused on patient and public involvement in the early stages (i.e., design and implementation), including projects focused broadly on the patient experience, or narrowly on specific population groups or specific areas of research [9–13]. More recently, several journal articles describe frameworks for how to integrate patient and family advisors in all phases of the research process and include specifics of how to share decision-making between researchers and other stakeholders [14–16]. Others describe the training and competencies of researchers required for effective stakeholder engagement [16, 17]. A review of stakeholder engagement highlighted that the evidence of impact was weak due to inconsistent data and lack of detail [3]. Studies to date emphasize the importance of evaluating the process of involving patients and the public in all phases of research and the need for evidence of how stakeholders’ perspectives can be meaningfully synthesized and used to shape research design, implementation, and dissemination [3, 10, 18–20].

This paper describes how stakeholders contributed to the design, conduct, and dissemination of findings of a multicenter pragmatic clinical trial and the changes the academic team made to the study in response to their input. The cluster-randomized COMprehensive Post-Acute Stroke Services (COMPASS) Study investigated the effectiveness of implementing an evidence-based, comprehensive, post-acute stroke transitional care model compared with hospitals’ usual care. The study design and methods – including methods used to engage stakeholders – are published [21–23]. This paper describes how stakeholders shaped this multicenter pragmatic trial.

## Materials and Methods

### COMPASS Study

The COMPASS Study was a pragmatic cluster-randomized controlled trial conducted in 40 hospitals with approximately 10,000 stroke and transient ischemic attack (TIA) patients in North Carolina. Intervention hospitals employed a novel transitional care model, as well as an additional set of billing codes to provide financial incentives, in order to change provider behavior and increase quality of care and recovery outcomes for stroke and TIA patients. The study provided small supplemental funding to hospitals to offset research-related costs only. The primary trial results are published [23].

The COMPASS model was initially developed in collaboration with the Wake Forest Baptist Health Comprehensive Stroke Center clinical team. It incorporated their prior experience with stroke transitional care through the TRAnsition Coaching for Stroke (TRACS) Program and evidence from the most current scientific literature [24].

**Fig. 1.**
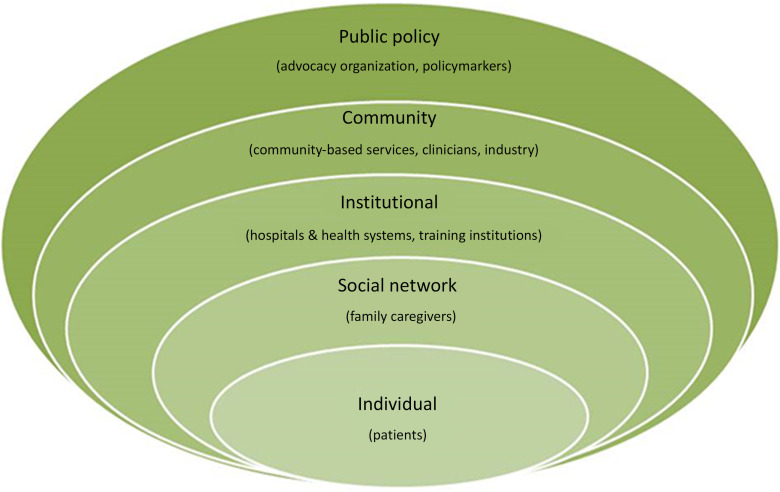
Stakeholder groups representing all levels of influence on patient health participated in the COMPASS study during design, conduct, and dissemination ^[21]^.

Details on the COMPASS Study’s non-traditional research partners and the rationale for their inclusion have been published [21]. Briefly, the study team included members from a wide range of stakeholder groups, representing all socio-ecologic spheres of influence on patient health, including: patients, caregivers, clinicians, community-based health and social services, hospitals and health systems, industry partners, advocacy organizations, payers, and policymakers **(**Fig. 1 **)**. Our primary goal for stakeholder engagement in the COMPASS Study was to be responsive to both patient and caregiver needs, to treat recovery from stroke as a chronic condition, to create a realistic workflow for hospital-based providers, and to link patients to community-based services to address social determinants of health to maximize recovery.

### Data Collection

We used qualitative methods to investigate stakeholder needs and priorities, which enabled us to more fully capture insights and reactions than would be possible with quantitative methodologies with pre-defined response options. Qualitative methods also allow new areas of inquiry to emerge and can reveal perspectives that researchers may not be able to foresee [25]. Transitional care is a process in which the patient moves from one healthcare setting and set of providers to another. During this transition, providers do not typically understand the next step in the care chain or the barriers patients face; the patient is the only “stable factor” in this process and thus the best source of information. Thus, qualitative methods were ideally suited for our purposes of improving the delivery of transitional care from the patients and multiple providers’ perspectives and within the clinical workflow.

Eliciting and incorporating stakeholder perspectives involved embedding stakeholders into study committees, assembling resource networks, conducting focus groups, and group discussions. These group interactions encouraged stakeholders to talk to one another, ask questions, and comment on one another’s experiences and perspectives, leading to more nuanced recommendations. Furthermore, the iterative process of holding multiple discussions over time with diverse groups of stakeholders helped refine recommendations and confirm that recommendations correctly reflected their ideas and were feasible with existing resources.

We collected input from stakeholders using committees and resource networks, as described below.

#### Statewide patient and stakeholder engagement committee

At the state level, we formed a large group of partners to be both inclusive and to leverage the tremendous expertise across the state. We purposefully included diverse perspectives, including urban/rural lived experiences of patients and providers, as well as patients and caregivers who represented varied income and educational levels. Some stakeholders on this committee were embedded in the study’s Steering Committee and subcommittees, which met weekly, so that they could have continuous input throughout the study. Some stakeholders were consulted outside the regular meeting schedule, most frequently by the study’s Principal Investigator (PI) and the Director of Implementation, both clinicians with extensive familiarity with North Carolina’s stroke system of care. Other stakeholders provided important insights via focus groups and interviews. See Supplement 1 for an example of what one focus group of elder, rural residents revealed to researchers, how that information was used, and how these stakeholders were informed that we had made changes to the study in response to their feedback.

#### Community resource networks

At the local level, we guided the post-acute care coordinator at each of the 40 participating hospitals to develop a COMPASS Community Resource Network (CRN). These networks of community-based health and social service providers helped the post-acute care coordinator link patients to resources outside the hospital that could support recovery. The goal was to incorporate local knowledge into the intervention to maximize successful implementation. Each CRN included a representative from aging services, a pharmacist, a home health provider, and a rehabilitation provider. Some CRNs also included a representative from the local health department, a community paramedic, a faith leader, a local stroke survivor, a local caregiver, a transportation service representative, and/or a social worker, reflecting each community’s unique resources and strengths. As part of implementation, each CRN engaged with the study’s Director of Implementation and other implementation and training team members during on-site day-long hospital meetings at the beginning of the study. CRNs also provided feedback to the study team by participating in bi-weekly group conference calls for problem solving. CRNs also participated in two surveys in which they shared challenges they had experienced as well as solutions. These data collection activities allowed the study team to continuously learn from those delivering the intervention.

### Consent

All stakeholder engagement activities were submitted to the Wake Forest University Health Sciences Institutional Review Board (IRB). The IRB classified (and approved) some stakeholder engagement activities as “Human Subjects Research” and other stakeholder engagement activities as “Not Human Subjects Research.” For IRB-approved research activities (focus groups, interviews, surveys, recorded group conference calls), participants provided verbal consent. For activities that did not meet the IRB definition for research (working group meetings of the study team and stakeholders, day-long meetings to discuss ideas with stakeholders, documentation of decisions made by study team members after discussion of stakeholders’ ideas), there was no consent process.

### Data Analysis

Guided by Grounded Theory, we asked stakeholders about their unmet needs, priorities, experiences with, and recommendations for post-acute stroke care. We extracted themes which were discussed as a team and with other stakeholder groups for feasibility. A consensus approach was used [26, 27]. Due to resource constraints and the intention of including stakeholder feedback continuously (starting with proposal development and throughout the 5-year study period), the notes from the focus groups, interviews, surveys, and meetings were not tagged with codes. Illustrative stakeholder quotes are included to support selection of themes. Stakeholder ideas that were intentionally incorporated into the study were documented in real time using a tool we developed with the REDCap software system. This tool (Stakeholder Engagement Tracker) is available in the REDCap shared library [28]. Throughout the study, we documented how stakeholders’ ideas and input shaped and refined the study using pre-defined data fields. These data fields were selected from PCORI’s reporting requirements, to document which stakeholders were involved, how, during which phase of the study, what the advice was, and whether it was incorporated. This process allowed us to search the database. Open-ended data fields captured details (see Gesell *et al.* 2020 for description of tool) [28].

We followed the Standards for Reporting Qualitative Research (SRQR) reporting guidelines, the completed checklist for which is available in Supplement 2.

## Results

With exception of development of the statistical analysis plan and the conduct of the statistical analyses, all aspects of the study were shaped by inviting and addressing the perspectives of non-traditional research partners. This engagement resulted in important changes to many aspects of post-acute stroke care. In addition, stakeholder input modified training for caregivers who are essential for stroke patients’ recovery, for hospital-based providers who generally lack awareness of what patients deal with after they leave the hospital, and for community-based health and social service providers whose services can support recovery yet often go underutilized because they are not integrated with hospital-based care and are unfamiliar to the patient. Details on the diversity of participating stakeholders, as well as key lessons learned from each group, and the ways that they shaped the study are presented in Table 1. Also described are ways that stakeholders’ engagement with the academic team, in turn, informed changes within partner organizations that extend beyond the scope of the trial.

**Table 1.** How stakeholders influenced the COMPASS study and the COMPASS study influenced stakeholders (examples)

Stakeholders	Diversity of stakeholders	Lessons learned from stakeholders	Stakeholder influence on COMPASS study	COMPASS study influence on stakeholders
**Patients** (Individual)	Given the profound inequalities in stroke outcomes, we intentionally promoted health equity by including the voices of female, African American, and rural North Carolinians as our lead patient partners.	At discharge, patients felt overwhelmed. Patients wanted care post discharge to include: 1. A follow-up phone call. 2. The name and telephone number of a single person who could answer questions. 3. A “roadmap to recovery” to maximize their recovery and prevent another stroke. 4. Doctors to tell them how important rehabilitation therapy is for full recovery and that recovery from stroke is a process that can often take many years. 5. Help managing medications at home. 6. A directory of resources made available to the public. 7. Help for families arranging social and medical services after discharge. 8. Support for caregivers because patients depend on them.	Patients shaped the design of the intervention to include: 1. A follow-up phone call from the hospital to ask what problems the patient is encountering at home in the first two (2) business days after discharge. The time frame was set to align with existing CMS transitional care management billing codes. The nurse could refer patient to home health or outpatient rehabilitation, if indicated. 2. The name and telephone number of the stroke care coordinator at the hospital who the patient or caregiver could call with questions, printed on a refrigerator magnet. 3. The magnet also depicted what patients could expect from the intervention (2-day call, 14-day clinic visit, care plan, connection to community-based resources, 30-day call, 60-day call). As part of the 14-day clinic visit, an individualized care plan with an uplifting and hopeful tone was developed with the patient and caregiver and shared with all providers. 4. Care teams were trained to emphasize the importance of rehabilitation therapy and how to assist patients with exploring ways they can access and afford needed therapies. Patients also helped develop patient handouts on rehabilitation and insurance barriers (See Toolkit for Patients and Caregivers at https://www.nccompass-study.org/). At times, we used the exact language given to us by patients. For example, in the Movement Matters handout, providers are clearly directed to “Inform your client: Recovery is a process that can take years.” 5. The pharmacist on the study team developed a medication reconciliation and adherence toolkit collaboratively with stroke neurologists and primary care physicians (see https://www.nccompass-study.org/). 6. The most comprehensive Community Resource Directory to support stroke recovery was built to populate care plans. It is also publicly available, and search functions have been revised based on patient feedback. https://www.nccompass-study.org/patients-and-caregivers/resource-directory/. 7. Most care plans focus on the patient’s needs, without recognizing the significance of the caregiver in recovery. The COMPASS care plan also includes the caregiver’s ability to care for the patient and, when indicated, connects them and families to community-based resources for support. 8. The hospital recruitment brochure featured verbatim quotes from patients to illustrate to hospital administrators and clinicians existing gaps in care and how the COMPASS care model was responsive to patients’ needs after they were discharged from the hospital. 9. Patients helped train interventionists by educating hospital-based clinicians across the state on what it means to deliver patient-centered care. 10. The primary outcome measure was the Stroke Impact Scale-16 because it was developed with input from patients, caregivers, and clinicians, and it captures what is most important to patients after stroke – physical functioning which allows for independent living (32–34). Before outcomes were collected by professional interviewers, patients instructed them to read questions much more slowly than they were accustomed to doing so that stroke survivors could understand and answer the questions. Patients also asked for visual cues to be added to the paper surveys so that respondents could more easily follow along during the interview. While we did not test different approaches, we speculate these stakeholder-informed changes to data collection improved data quality.	As the study team listened to and learned from patients, patient-facing, evidence-based materials and resources were developed at a sixth grade reading level and are now available on the COMPASS website for use not only by patients but also by health and human service agencies that serve this population. They are free of charge and downloadable in English and in Spanish. They are also included on the NC Department of Health and Human Services Start With Your Heart website as part of the NC Stroke System of Care Plan.
**Family Caregivers** (Social Network)	We sought input from families who represented varied income and educational levels and rural/urban locations.	1. All caregivers are overwhelmed and do not know how to care for their family members after discharge (even the highly educated with access to resources). 2. Caregivers said they had to teach themselves how to communicate with patients with aphasia and do not feel like providers are sufficiently trained to do so either. 3. Patients are discharged home with overwhelming amounts of paperwork and often with cognitive deficits that are not detected in the hospital.	Caregivers influenced the study design: 1. Caregivers decided on the timing of study consent. Caregivers clarified that the most convenient time to consent patients from a health system workflow perspective was the worst time to expect a patient with stroke to understand consent. 2. Caregivers overhauled the consent form language by working closely with researchers and the IRB to make sure the form would not distress patients in the control arm of the study. Caregivers worried that patients in the control arm would think that they would be receiving substandard care, and that that might interfere with their recovery. We arrived at a consent form that clearly explained randomization yet did not raise concerns. Together the consent process informed by patients and caregivers reached the highest standards of respect for persons, rather than simply meeting the minimum IRB requirements (35). Caregivers influenced implementation: 3. Caregivers educated hospital-based clinicians on why caregivers are the most important part of the care team and the need to involve caregivers in developing the patient’s care plan. In the care model, the care plan should be developed with the patient and caregiver. 4. A caregiver and former schoolteacher introduced the idea of training caregivers and providers to communicate respectfully and effectively to stroke survivors with aphasia and linguistic impairments. She developed the patient brochure based on content she had acquired while seeking to help her husband which was reviewed by a speech-language pathologist. Per her request, the speech-language pathologist developed a training video for providers. Providers were trained on the video and how to present the patient brochure to their patients. 5. All intervention materials (care plans, educational brochures, etc.) were reviewed by stroke survivors and their family caregivers to make sure language was clear and that we captured what mattered to them. When we missed the mark, we made changes based on their feedback. For example, the tone and format of the individualized care plan was reworked in response to patients and caregivers saying it needed to convey a message of hope. All materials are available to the public in English and Spanish at https://www.nccompass-study.org/.	As the study team listened to and learned from caregivers and families, evidence-based materials and resources were developed at a sixth grade reading level and are now available on the COMPASS website for use not only by this group of stakeholders but also by health and human service agencies that serve this population. They are free of charge and downloadable in English and in Spanish. They are also included on the NC Department of Health and Human Services Start With Your Heart website as part of the NC Stroke System of Care Plan.
**Hospitals, Health Systems, Training Institutions** (Institutional)	Although the majority of the intervention occurs in the post-discharge setting, hospitals had to identify eligible patients and initiate the intervention before discharge. Thus, hospital-based stakeholders included: • All relevant members of hospital-based stroke teams (rural/urban): o Stroke neurologists o Stroke nurse practitioners o Stroke physicians assistants o Stroke nurses o Stroke program coordinators o Therapists • Physician emergency department directors • Quality/performance improvement coordinators • Case managers/social workers • Nurse managers • Patient education directors • Senior hospital administrators	1. Hospital-based stroke teams are aware of needing to provide value-based care and eager for guidance on how to do so in the post-acute stroke setting. 2. The potential barriers they identified during study design were later observed. 3. TCM billing is underutilized nationwide (7% in 2015 (36)). The hospital-based stroke teams had had challenges in the past using CMS transitional care management (TCM) billing codes because of: (a) uncertainty about how to deliver care that meets TCM billing requirements; and/or (b) conflict with primary care providers around which provider got to use the TCM billing code.	Multidisciplinary hospital-based clinicians influenced the study design: 1. These stakeholders made a key contribution to the study design by advocating for a delayed start design meaning that, at the end of the trial, all hospitals enrolled in the study eventually received the intervention. This study design was chosen to support hospital recruitment and prevent drop out during the trial. It requires twice the effort and cost but was critical for hospital buy-in, recruitment, and retention. Multidisciplinary hospital-based clinicians influenced implementation: 2. The intervention required new structures and processes of care be put into place at each hospital. The first step was to add a post-acute care coordinator to the clinical team. Stroke program managers across the state co-wrote the job description. Once they understood what the research team wanted this person to do, they were better equipped than the academics to articulate the training, skills, and traits necessary for this role. They also reset the study team’s expectations for what hospitals could realistically find in their local workforce. 3. Barriers predicted and then observed included difficulty completing a follow-up visit with 100% of stroke patients in hospitals with a large volume of stroke patients. While TCM billing requires a face to face clinic visit in 14 days, the study protocol was adjusted to 30 days. 4. We provided education on TCM billing mechanisms so that hospitals could successfully get reimbursed for the intervention. This proved to be insufficient until first wave post-acute care coordinators shared how they had also used alternative extended care codes to avoid tension among providers even within their own health system. 5. The first wave of experienced post-acute care coordinators helped train subsequent waves of interventionists. They provided initial and ongoing peer coaching on how to set up a COMPASS clinic, challenges to anticipate, how to address them most effectively, and in some instances, even how to avoid them altogether. 6. We made changes to the electronic application designed for delivering the intervention in the study to address workflow concerns voiced by clinicians after they tested the application and how to administer it to patients on an iPad. The hospital-based stroke team influenced data quality: 7. NIH Stroke severity scale (NIHSS) scores are entered into patient charts as part of usual care, although nationally 44% (37) are missing from charts. Once the clinical team understood the importance of documenting these scores, and then once the research team understood the challenges with abstracting the NIHSS scores from the patient charts, the study teams nurses and physicians produced training materials for retrospective data collection of NIHSS scores. After this training and technical assistance was launched, the missing NIHSS data element for all Phase 1 enrolled cases was reduced from 31% (25 July 2016) to 2% of the enrolled cases (16 May 2018). In Phase 2, only 4% of the 4,237 enrolled cases had missing NIHSS scores (15 March 2019).	As hospitals and health systems across the state and nation learned about the collaborative nature of the COMPASS Model for providing care starting in the hospital and continuing in the post-acute phase, COMPASS team leadership was invited and/or offered to provide training on the model as well as to support hospitals and health systems interested in implementation of the model. This collaboration and the support of hospitals and health care systems outside of the 40 study sites allowed for an expanded reach. • The study team met one-on-one with 200 hospitals and rehab agencies to provide guidance and strategy for implementing the COMPASS model. • The study team is aware of 147 hospitals and rehabilitation agencies that decided to adopt the COMPASS model (or parts of the model). • The study team is aware of 110 hospitals and rehabilitation agencies that have sustained the COMPASS care model (or parts of the model).
**Community –based services** (Community)	These stakeholders are caring for stoke patients longitudinally and are essential partners for carrying out the care plan. We partnered closely with networks of: • Aging service providers • Caregiver support services providers • Outpatient physical, occupational, speech therapists • Home health agency leaders and frontline staff/teams • Primary care physicians • Pharmacists We made a concerted effort to include representatives of all groups from both rural/urban areas and from across the state because community resources vary dramatically by county.	1. Challenges connecting patients to social services remain. 2. Community health and social service agencies welcome hospital partnerships but have had significant challenges gaining access. 3. Hospital-based clinicians are not typically aware of the network of services available in the community. 4. Primary care physicians and pharmacists identified medication management as a modifiable risk factor, echoing patients and neurologists.	Community-based clinicians influenced the study design and implementation: 1. In response to patients and caregivers saying they needed help managing their medications after discharge, the pharmacist on the study team developed a medication reconciliation and adherence toolkit with stroke neurologists and primary care physicians. He then travelled across the state and met with pharmacists who were part of a statewide network of community-based specialty pharmacies (offering enhanced services), developed by the largest and longest-running medical home system in the US. Together, they developed an implementation strategy including how to consult with local primary care providers and refer COMPASS patients to local participating pharmacies. He discussed COMPASS during a CCNC webinar and encouraged the pharmacy directors from each CCNC region to be resource individuals for local pharmacists who have questions about the medication management intervention in COMPASS. The impact of this effort was a strong participation of pharmacists in the Community Resource Networks established at each participating hospital. These pharmacists then became critical partners to the post-acute care coordinators in caring for patients after discharge with expertise and resources for medication management, blood pressure management, diabetes management, smoking cessation etc. 2. A team of community-based service providers from different health systems (a home health provider, pharmacist, paramedic, aging support service care coordinator) trained post-acute care coordinators across the state on how to link their patients to existing, underutilized resources for recovery.	Since the inception of the COMPASS Model, then throughout implementation, the study team has worked extensively to break down silos among health and human service organizations. Large scale examples of this include: 1. The Director of Implementation for the COMPASS Study served on the planning team of a regional aging agency to plan for and execute a statewide conference on integrating human services and health care. 2. COMPASS leadership has been at the planning table for developing one of the lead pilot entities (LPE) in NC for Medicaid transformation.
**Advocacy Organization, Policymakers** (Public Policy)	Influential leaders involved in high level advising to support dissemination and sustainability included: • National advocacy organization • Regional networks of health and human service providers • NC chapter of national health insurance company • State legislators and stroke champions with influence on development and maintenance of NC’s stroke system of care	1. Patients and caregivers rely on advocates. 2. There is a large amount of evidence for the effectiveness of policies that reduce the risk for stroke. 3. Regulations and reimbursement for rehab/recovery services (e.g., neurology services for post-acute patients), and education/counseling to prevent stroke recurrence need promotion. 4. Support is needed for: • Stroke advocacy efforts • Policies to prevent stroke • Physical activity and healthy food promotion • Increased access to health care • State preventative care regulations and funding within and beyond Medicaid • Wellness visits related to stroke • Efforts to enhance stroke care and post-stroke care including telestroke equipment and services. • Secondary prevention education. • Funding for rural hospitals.	Public policy stakeholders influenced dissemination: 1. They developed and wrote the first draft of how to share results with the public and policymakers for maximum impact using constrained resources, leveraging their regional and national reach. 2. They have organized opportunities for the study team to keep state lawmakers (who influence hospital funding) informed of the study’s progress. 3. They have given academic researchers committee positions to inform and recommend critically important policy changes at the state level that facilitate improved stroke care.	COMPASS study leadership now serves on state and national committees, task forces, and ad hoc groups to share information about the needs of stroke patients and the needs of the caregivers, clinicians, hospitals, and community-based organizations who care for them after discharge. This has led to: 1. The development of the NC Stroke System of Care that spans the continuum of care. 2. Service on Stroke Advisory Council Work Groups has led to the creation of a NC Hospital Survey that now includes a post-stroke section to determine hospitals’ capacity to provide comprehensive stroke care across the continuum. 3. Service on the development team to assure that our region is in a good position to become a lead pilot entity (LPE) for Medicaid transformation in NC.

CMS = Centers for Medicare and Medicaid Services; COMPASS = COMprehensive Post-Acute Care Services Study; IRB = Institutional Review Board; NC = North Carolina; NIH = National Institutes of Health; TCM = Transitional Care Management.

Members of the statewide stakeholder committee added, shaped, and refined intervention components (e.g., the intervention assesses the *caregiver’s* ability to care for the patient) and all patient- and provider-facing intervention materials (e.g., content, language, tone, layout, timing of delivery to maximize understanding). Examples of these changes are described in more detail in Table 1.

Weekly Steering Committee meetings included a stroke patient, caregiver, hospital neurologist, pharmacist, nurse, rehabilitation therapists, stroke advocacy organization leader, community-based aging agency leader, and stroke and health services researchers. This diverse leadership team provided perspectives that were often different and sometimes even opposing – and resulted in better problem solving and products. For example, Steering Committee members leveraged their diverse networks to design a comprehensive dissemination plan that maximized reach, identified barriers, and used the most effective strategies to ensure timely and effective communication to patients, community leaders, hospital administrators, and policymakers. Based on stakeholder input, the final plan was written in plain language so that it could be understood by all.

Clinicians and senior hospital leadership identified barriers to attendance at protocol-specified follow-up clinic visits and identified ways to improve attendance at this critical visit. They also provided feedback on how to more seamlessly integrate the intervention into clinical workflows to improve institutional buy-in. In turn, this engagement resulted in dissemination of the COMPASS model in part or in whole to 147 hospitals and rehabilitation agencies beyond the trial sites, with 110 sustaining use. As one stroke coordinator shared with us,

“We had to learn the process of ordering things in the outpatient world instead of the inpatient world… it took us a long time to sort of get over some of those hurdles… in a year, we have actually transitioned I think because the practice and our administrators saw how wonderful this program was and how much we were helping our patients. They actually said “Wow!” … and they said, “we can give you one of ours [nurse practitioners] for half a day now.”

Additional examples of specific stakeholder input and feedback that we elicited through engagement during proposal development and how we modified the intervention and implementation strategies as a result are presented in Supplement 3. For example, patient and caregiver stakeholders shared ways that COMPASS providers could improve their communication with patients, such as repeating instructions and the *reasons* that certain recommendations were being made to increase adherence. As one patient stated:

“It was also important for the doctors and the therapists to explain it multiple times – not to assume I knew why I needed this.”

Also during proposal development, feedback from hospital-based clinicians alerted us to changes needed to improve communication with local primary care providers (PCPs). We added another PCP to our stakeholder committee and had him review all PCP-facing materials and the process of getting the patient’s COMPASS care plan into the hands of the patient’s PCP. During implementation, multiple hospital-based clinicians shared that they still encountered hesitation or resistance from PCPs who wanted to use transitional care management billing codes and viewed the study as competition for revenue or patients. This real-time feedback allowed us to improve our communications with both the PCPs and the clinical teams delivering the intervention and let them know of alternative billing codes (identified by the clinical sites, not the research team) and emphasize our goal of working collaboratively to improve the transitional care and outcomes of patients.

Importantly, by bringing together patients, community-based service providers and hospital-based providers, it became clear that there were gaps in care that patients could easily identify and that providers were unaware of but eager to address. As one stroke coordinator shared with us,

“[What] COMPASS has really done for us is identify some holes that are in our program and the speech therapy is a prime example… I didn’t know before starting COMPASS that these things were getting missed or they weren’t being done or the patients were struggling like they were…. You don’t know what you don’t know, and I certainly had no idea. So we have been able to work through and do some education and set up some processes where we can limit these missed therapies and missed opportunities for incorporating resources for the patient.”

The cross-sector collaboration in the study also gave community agencies an entry point into their local hospitals which, in the past, they had not been able to establish on their own. There were multiple points throughout the study in which these stakeholders drove engagement activities that clearly strengthened the study. For example, the study pharmacist saw opportunities, knew of an existing network of community-based pharmacies that offered enhanced services, and had critical personal connections that made it possible for the study team to link a community-based specialty pharmacist to each of the 40 hospitals (via their CRNs). These community pharmacies often were able to provide to patients evidence-based interventions (smoking cessation, diabetes management, blood pressure monitoring, etc.). The study team recognized the value that these community pharmacists offered, but, over the course of implementation, they emerged as markedly more critical partners in care than the team had initially expected. If a pharmacist had not been on the study team, the team would not have been aware of the opportunities he identified; nor would the team have had the credibility or social capital that he had in his professional network to forge these statewide partnerships and connect patients with community services to support their recovery. Expanding beyond the hospital perspective enabled us to treat the patient holistically.

The study team made several observations after including diverse stakeholders in relation to socioeconomic status, urban/rural location, and racial representation. First, regardless of socioeconomic status, location, or race, patients and caregivers voiced the same problems with usual care after stroke:

- Not knowing who to call as soon as they recognized they had cognitive and/or physical deficits that were not detected in the hospital.
- Survival and full recovery require that the patient has an able and willing caregiver, yet many stroke survivors live alone or have ailing spouses who themselves need caretaking.
- Not having a roadmap to follow to recovery.

These unmet needs were universal and shaped the intervention. It was particularly eye opening to hear from highly resourced patients and caregivers that they – in spite of their social and economic advantages – were overwhelmed by having to managing post-acute care. They told us that they did not see how people with fewer resources could cope or ever fully recover. Their lived experience pushed the team to design an intervention that assesses the patient comprehensively, and *also* assesses the caregiver’s ability to care for the patient. The intervention also calls for a single person, trained in stroke, to be the clear contact person for the patient; and this information is communicated to the patient and/or family in a myriad of ways (introduction at bedside, business card, refrigerator magnet, in paper work, by mail, second introduction on follow-up call after discharge). The guidance stakeholders gave for a roadmap to recovery (Care Plan) is described in Table 1.

Second, stakeholder diversity in terms of rural/urban and racial representation produced creative solutions to common problems. Rural hospitals had clinical teams that were exceptionally effective at anticipating and successfully addressing implementation problems. They attributed this prowess to knowing most people in the small hospital and community and being able to leverage those relationships. The solutions developed at rural sites were shared with and then adopted by urban sites. For example, a rural site could not identify the protocol-specified RN within the hospital system to serve as the main contact to the patient. But a clinician did know of a community paramedic program, rooted in the African-American community that was already making home visits. The site argued that a community paramedic, who was trusted by community members, could effectively function in the protocol-specified role and the protocol was amended. This solution was then used in other sites.

Several clinical stakeholders who were African-American and started as advocates for their African-American patients became trail blazers in implementing adaptations of the intervention after the measurement period (during the sustainability phase) to further address their needs. For example, to address remaining transportation barriers, they consolidated members of the care team to one location, and made the comprehensive assessment of the patient a televisit. The study team’s next study is to test the effectiveness and implementation of a televisit vs in-person visit for comprehensive post-acute stroke care now that Medicare is covering telemedicine during the COVID pandemic and this form of the intervention is sustainable.

## Discussion

In this study, we actively elicited and incorporated input from a diverse set of stakeholders in one of the first large-scale pragmatic clinical trials of transitional care in the USA. This qualitative assessment highlighted ways that patients and caregivers shaped the design of the intervention, enhanced caregiver and clinician training, designed ways to improve patient recruitment and participation, and made patient-facing materials more useful to other stroke patients and their caregivers. Furthermore, it revealed ways that engagement of acute care hospitals’ providers and administrators, post-acute care providers, local pharmacists, and primary care physicians facilitated uptake of a complex intervention. Unexpectedly, it also revealed ways that stakeholders themselves benefited from engagement with the research team.

The majority of the literature to date on stakeholder engagement in research has provided general guidelines or has focused on the barriers to successfully engaging stakeholders [29]. A common conclusion in these studies is the need for real-world evidence of successful stakeholder engagement methods. Findings presented in our paper describe specific ways that active engagement of a broad-ranging and diverse set of stakeholders shaped a large-scale pragmatic clinical trial. While the majority of studies that have shared successful engagement practices have primarily focused on only one aspect of the study (e.g., design, implementation), this study describes ways that a complex intervention bridging acute and post-acute community settings was shaped by stakeholders throughout its design, conduct, and dissemination.

Patient and family stakeholders were typically delighted when asked to join or advise the study team. Indeed, many patients sought out the study team when they heard about it through the local media. Patients and caregivers were typically motivated to collaborate as partners when learning that the research team wanted to learn from their experience to improve care for others. Community-based health and social service providers were similarly motivated to serve as stakeholders, even those who had no prior research experience, due to the shared mission of improving health and wellbeing of either stroke patients in particular or older individuals more broadly. Patients were open and honest with their input and feedback. At times, patients and families would couch their critiques of the hospital care they received by explicitly praising their providers (paraphrasing: “I loved my nurses, but xyz has to change”). Patient and family stakeholders frequently expressed that being able to contribute to the study was critical to their own healing. Specifically, they stated that bringing awareness to gaps in care and being part of the change that they hoped would reach thousands of other patients helped them rebuild their confidence in public speaking, redirect their grief or anger into something productive, make sense of their new life, and fulfill their new purpose in life.

This paper has limitations. Due to resource constraints it was simply not feasible for the study team to deeply engage with all stakeholders and also transcribe and tag all data with codes. Engagement activities were tracked but some were captured in less detail, truncated or summarized in REDCap forms. However, in those cases we often uploaded documents that captured rich details about the engagement activities. The strength of this paper is that the team documented the vast majority of engagement activities and systematically engaged stakeholders from design to dissemination and acted on stakeholder advice.

Stakeholder involvement in the COMPASS Study produced lasting benefits to stakeholder groups beyond the scope of the study, from publicly available, patient-friendly educational materials (https://www.nccompass-study.org/), to education of hospital-based clinicians on what it means to deliver patient-centered care, to strengthened community partnerships, to sharing evidence that informed policymakers about the need for primary and secondary prevention care in stroke. Very importantly, stakeholder involvement in the study produced a new patient-centered consent model for future pragmatic trials [30].

Inclusion of stakeholders from underrepresented groups enhanced the project overall. For example, working with an African-American patient stakeholder on the development of the consent model was extremely educational. While she was advocating for all stroke patients, she was particularly attuned to the deep mistrust of the health system that African-Americans in North Carolina still feel. She advocated for a delay in the timing of consent (ensuring that patients were not asked to consent when they could not fully process what was being asked of them). She changed the language used on the consent form and study brochures because she did not want a single patient to think they would be receiving suboptimal care if assigned to the control group (for fear that perception would undermine their recovery). While the study team could offer language the IRB approved, she could offer language that would not turn patients away from the study.

A common charge against stakeholder involvement is the amount of time and resources required. We agree that, for stakeholder engagement to be meaningful, it does require paid research effort, study infrastructure, and payment for the time that patients and community organizations provide to the study. The study budget was established in 2015. It was important to reimburse stakeholders equitably and consistently. See Supplement 4 for our policy and procedures for reimbursing stakeholders. The study paid $20 per hour to patient and caregiver stakeholders for their time spent preparing for meetings, attending meetings, reviewing materials, and travel when necessary, etc. For health professional and corporate stakeholders there was overlap between their established (paid) work and study needs, therefore they were not compensated at an hourly rate. All stakeholders were eligible for compensation for their time in focus groups and interviews which the IRB defined as research. It was necessary to balance compensation across a large stakeholder base while not limiting input from stakeholders or exceeding study budget. It is now 2020, and as our team prepares other grants and associated budgets we have increased the hourly rate to 30–40 dollars per hour (depending upon the size of the grant) as we feel that 20 dollars per hour should be increased for the value that stakeholders bring to each study.

It should be noted that a collaborating advocacy organization declined reimbursement. A community-based organization negotiated a 10,000 dollar per year flat rate. Reimbursement went to the organization, not to the individuals engaged in the study. Unlike community-based participatory research, stakeholder engagement in research can lie along a continuum of meaningful participation, which provides some flexibility in time and resource investment. We believe that the short-term costs of investing in stakeholder engagement pays off over the long term. For instance, it may take longer to create a consent process and consent form that patients, caregivers, and the IRB approve, but it may support patient recruitment and hospital retention over the course of the study.

Future studies should directly test the impact of elements of study design, implementation, and dissemination that were informed by stakeholders vs those that were not to continue to advance the science of engagement in clinical research. Future research should also quantitatively test the effect of stakeholder engagement on patient recruitment, patient or provider behavior, and patient-centered outcomes. Another area ripe for study is understanding how patients personally benefits by contributing to research projects (not as subjects, but as study team members) and if and how that might affect their own recovery. This will require additional time and resources but is critical to strengthening the inclusion of stakeholders in the design and implementation of future studies and programs.

## Supplementary material

For supplementary material accompanying this paper visit https://doi.org/10.1017/cts.2020.552. click here to view supplementary material
