# Supplementary material for: How engagement of a diverse set of stakeholders shaped the design, implementation, and dissemination of a multicenter pragmatic trial of stroke transitional care: The COMPASS study
Source: J Clin Transl Sci. 2020 Nov 5;5(1):e60. doi: 10.1017/cts.2020.552 (PMC8057438; doi:10.1017/cts.2020.552)
Supplement: Supplementary file 1 [file S205986612000552Xsup.zip › S205986612000552Xsup002.docx]

**Multiple staff from 29 stakeholder hospitals participated in webinars to discuss potential barriers of successful implementation of the COMPASS model and solutions.** Participants included stroke program coordinators, stroke neurologists, physician emergency department directors, quality/performance improvement coordinators, nurse managers, patient education directors, hospital administrators, and other members of hospitals’ stroke and administrative leadership teams. Using the NCSCC’s existing infrastructure, we conducted 6 webinars June 9-18 to introduce the COMPASS model and discuss its application within their unique settings.

**Participants stated that they expect the COMPASS model will benefit patients by:**

1. Increasing compliance with prescribed treatment that will reduce their risk of recurrent stroke and other problems, while improving recovery.
2. Providing resources of navigators not currently available in rural communities.
3. Imposing a structure to ensure patients are being managed across the continuum; hospital participants said they would like to provide this cross continuum care but they are not.
4. Connecting patients to community resources.
5. Providing resources for rapid follow-up rather than two months after discharge.
6. Monitoring patient outcomes.
7. Having a designated Navigator coordinating care and advocating for patients.
8. Creating a network and forum for clinicians, administrators and community providers to share best practices across the state to improve the quality of post-acute care.

**Participants stated that they expect the COMPASS model will benefit the health system by:**

1. Enhancing patient care without disrupting current hospital systems and programs.
2. Providing educational opportunities with CE credit.
3. Funding the nurse Navigator Role to be the community/patient resource and engaging hospitals and practices to utilize the TCM codes.
4. Developing a post-acute care system of management for complex patients like stroke. Testing a model that might be beneficial for other diagnoses as well.
5. Improving patient satisfaction and perceptions of care through continued involvement after discharge and better continuity of care across the continuum.
6. Developing and communicating a disease management care plan aligns everyone to accomplish better patient engagement.

**Participants identified the following potential barriers to success:**

1. Hesitation or resistance from primary care providers (PCPs) who want to use TCM billing codes and may view this study as competition for revenue or patients.
2. Difficulty identifying qualified NPs/PAs and Nurse Navigators to be part of the COMPASS team, especially in rural communities.
3. Potential for excessive workload for NPs/PAs operating in rural hospitals serving patients from multiple counties.
4. Difficulty completing 7-14 day follow-up visits for 100% of patients in hospitals with large volume of stroke patients.
5. Lack of past success with private insurance and Medicaid reimbursing TCM codes.
6. Data capture and data reporting requirements for this study may require changes to existing data systems.
7. Limited access to primary care in rural communities.

**In response, we have modified our intervention and implementation strategies and will:**

1. Include a PCP as a key stakeholder involved in the statewide Patient and Stakeholder Engagement Committee and actively manage relationships with PCPs during the trial to ensure they feel represented and supported by the COMPASS model of care. *(Note: Pushback from the PCP community is unlikely, as PCPs typically do not have the time to meet the TCM billing code requirement of a minimum of 45 minutes of care coordination for complex patients like stroke patients. The COMPASS model will support PCPs by stabilizing patients after discharge and linking patients without medical homes to their practices. If PCPs do have nurse navigators in place, the COMPASS nurse navigator will work as a back-up to care coordination.)*
2. Help hospitals identify qualified NPs/PAs and Nurse Navigators.
3. Use a multiple NP model in sites that pull from large catchment areas and with high volumes.
4. To guide quality improvement (QI) efforts, provide quality metrics back to hospitals—including the proportion of patients who receive 7-14 day follow-up visits—with comparative data from other participating sites in aggregate.
5. If funded, we will include payers in the statewide Patient and Stakeholder Engagement Committee to help ensure reimbursement for TCM codes. WFBH has already developed innovation projects with BCBS of North Carolina, to improve management/support reimbursement of complex patients and we will bring this program forward to them if funded.
6. Provide technical support for data abstraction using the NCSCC infrastructure, similar to what has been offered in previous acute care QI efforts.
7. Include NC Area Health Education Centers (AHEC) collaborative, NC Area Agency on Aging (and other community supports to ensure a strong network of community providers and community services and to enable a strong working partnership in the community).

**60 year old, African-American female, living in urban NC (stroke at 45)**

It is difficult after a stroke. It is difficult reaching for resources. It is difficult communicating. It is difficult caring for yourself if you live alone. Seniors are becoming caregivers. They are weak and frail themselves and then end up becoming patients because they are taking care of siblings, husbands. It is difficult for everyone in the family. There is a real, real need for assistance when patients get home. You can’t just place an individual back in their home with a bottle of pills and a follow up visit. I am not a clinician, I just know what I needed; and I know what other stroke patients need.

***“I am not a clinician, I just know what I needed; and I know what other stroke patients need.”***

I wish there had been a clinic in place, one that I could have called as soon as difficulties started. Someone to answer questions. I wish there had been follow-up calls. Someone to ask me if and when I was taking my medications, advising me to keep a record. Someone to ask me about my diet, advising me to keep a record of my diet. You may think it is not possible to do something like this, given how expensive it is to receive medical care, but with the care system as it is, I think it is not about more dollars and cents, I think it is about revamping how clinics address patient needs.

I started a support group for stroke survivors. The main challenges we discuss are medication, diet, exercise, and insurance issues that are keeping them from seeing a doctor. People bring their medication bottles in because they don’t understand them. They ask me about interactions. Of course, I have to refer them back to their doctor. But this is sad. It points to a breakdown in doctor-patient communication. Obviously they would talk to their doctors if they felt they could. I am so tired of hearing survivors say they can’t have these conversations with their doctors. I tell them they need to go in prepared. And they do, but the doctors don’t have time to answer their questions. I asked one gentleman if I could accompany him as his advocate. Now this gentleman can speak for himself, very well in fact. After the doctor checked him and asked him about his medications he turned to his computer to type. I asked the physician if Mr. X could ask him some questions while he was typing. You know what he said? “Make if fast.” Mr. X asked a simple question about his medications and diet. He felt that he was having a reaction after eating certain foods. Then the doctor gave an answer any guy on the street could have given. “If it is not making you feel well, don’t eat it.” Then he excused himself and went to see his next patient. This is not unusual and a common problem I hear from patients all across North Carolina. Doctors are hung up on managing time. So what is in place for the patient? Nothing. Seriously: nothing. No visiting nurse, no one to answer questions, or help them get what they need. That is why people end up back in the hospital.

***“So what is in place for the patient? Nothing. Seriously: nothing. No visiting nurse, no one to answer questions, or help them get what they need. That is why people end up back in the hospital.”***

I want to see a program to meet the needs of the new patient. We have so many questions: How to access systematic assistance, how to be a caregiver, how to access tools I need. Is there a hotline we can call? And I don’t mean one where they make you hold for hours and still don’t answer your questions. Someone with answers. Someone you can talk to and say: “I can’t afford a nursing home. Who can help me get a lift for my husband? I know there is a prescription drug program. Who can help me access it?”

**31 year old, white female, living in rural NC, high school graduate, associate’s degree (stroke at 22)**

***“It was also important for the doctors and the therapists to explain it multiple times—not to assume I knew why I needed this.”***

I was 22 years old when I had my stroke. I had a 2 year old and a new baby at home. I never thought something like this could happen to someone my age. I was treated at Forsyth Hospital and the biggest thing I remember was the doctor’s pushing for me to go to therapy. They stressed it was an important role in my recovery and being able to gain back my independence. It was so important for the doctors to explain why I needed to go to therapy and to use simple terms so that I could understand what was going to happen. With my brain not working properly, it was important to have things explained slower and in smaller, non-medical terms. It was also important for the doctors and the therapists to explain it multiple times—not to assume I knew why I needed this.

Once I got home, I had a lot of mixed emotions about what to expect and how my life would be now. A vital item they gave me at discharge was a book on stroke. This book helped me so much and explained the side effects from stroke—what to do and what not to do; the symptoms to watch out for in case I had a second one; how I would feel—both physically and mentally; and even covered sexuality after a stroke. This was huge to me as a young woman!

My family was a huge support in my recovery. My husband had to go back to work shortly after so my mother and sister stepped in to help care for me and my kids. I attended rehabilitation at Hugh Chatham. My mother and sister would drive me back and forth to rehab—an hour and 10 minutes round trip, 5 days a week, for months. They would then watch my children while I attended therapy. My stroke was on the left side of my brain which mainly affected my speech. The therapist would have me read Clifford books and it was so frustrating because my brain new what to do but my mouth couldn’t get the words out. I also attended occupational and physical therapy because the stroke also affected my balance. Not being able to balance or speak was so frustrating because it made me feel like I was dumb. And as a young mother with 2 small kids at home, it was so frustrating to not be able to care for my children or even hold my newborn baby.

***“Stroke is just as hard on family members. They carry a large portion of the weight of recovery.”***

***“The biggest thing that helped me recover was that the doctors, the therapist and my family all pushed to make sure I went to rehabilitation. If it had been up to me, I would have given up and quit.”***

Stroke is just as hard on family members. They carry a large portion of the weight of recovery. The biggest thing that helped me recover was that the doctors, the therapist and my family all pushed to make sure I went to rehabilitation. If it had been up to me, I would have given up and quit. It is important to have determination. I was surprised at how quickly things came back to me. Most of the time when I had heard of someone having a stroke, it meant that they died or were paralyzed for the rest of their life. I didn’t know this type of recovery was possible!

**60 year old, white male, living in urban NC, member of the business community**

I had a midbrain ischemic stroke 6 years ago that left me with the inability to swallow and complete aphasia; I could not speak. After 3-4 days I was released home with some cognitive deficits but virtually no physical or occupational deficits. I could function, I could communicate slowly. People were patient as I searched for words; but because I looked fine—I didn't have one sided paralysis—they thought I was fine. I am a board member of the North Carolina Stroke Association and on the board of Onslow Memorial Hospital here in Jacksonville, NC. At the time of my stroke I was just a regular patient, I did not have any connection to the hospital. But now if there is one thing I advocate for in these roles, it is for a navigator or ombudsman for stroke patients.

***“If there is one thing I advocate for, it is for a navigator or ombudsman for stroke patients.”***

I knew my local pharmacist and they knew my prescriptions. I had had a heart attack previously. After the stroke I had new prescriptions. And this was the first obstacle for me: I couldn’t dispense my medications into daily doses. The math required to do that was beyond my ability. This math deficit was not recognized until I got home. I lived alone and I had to take care of myself and I was unable to cope. The simple act of maintaining post care medications was extremely difficult for me. I know the nurse explained my medications to me at the hospital. If you had asked me if I received discharge instructions I would have checked the box ‘yes’. I could hear the discharge information but I had no idea how it would manifest itself at home. In the hospital I could show I could give myself an injection so that I could take anticoagulants at home. But my medications needed to be interpreted in terms of dosage and frequency. I found help with these pieces and worked through it. But a phone call check-in from a navigator or ombudsman to ask what problems I was encountering at home that first day or the next morning at the latest would have been a tremendous benefit. A follow-up phone call has got to be the prime piece that has to happen in stroke recovery. Just like there are follow-up calls after surgery. When you are taken out of the hospital setting you just take a deep breath and think ‘Maybe I can get through this…’ and then you hit obstacles; the anxiety is so tremendous you almost regress. One to three check-in calls in 30 days would be a godsend.

***“This math deficit was not recognized until I got home. I lived alone and I had to take care of myself and I was unable to cope.”***

***“A follow-up phone call has got to be the prime piece that has to happen in stroke recovery.”***

My rehab was the best. I was blessed. I had good local therapy. I had the right therapist at the right time. She gave me the best care humanly possible. She worked me ruthlessly. She didn’t let me stop working because I told her that is what I wanted. She was brilliant and she was loving. She challenged me and I competed with her to get back to where I am now. I bought math flash cards to practice at night and on weekends so I could go back to rehab and show off my progress. I am an insurance agent so I knew exactly what services insurance would cover. But I asked for extra rehab. It was invaluable to me. I signed up for every open slot and paid for it myself. Costs from everything else were excruciating. I was just getting out from under the costs of the heart attack I had had too. Rehab is the only reason I can sit here now with the only deficit in proper names. For people with physically debilitating strokes—like my mother—physical therapy and speech therapy are the only things that mattered in her life until the end. I was blessed with the ability to access good local rehab because you can’t drive and hour and do short-term rehab—that is no good to a person. It needs to be in your community.

I think I was spared so that I could tell my story. That is how I am looking at it now. I was extremely excited to hear this interview is tied to a grant proposal. You are right on point. I am delighted to be involved. We are going to save lives—not like a surgeon would—but we are going to save people's ability to have a life.

**60 year old, white female, living in urban NC, high school graduate, some college**

I was in the hospital for 2 ½ weeks after my stroke and while in the hospital, I only remember bits and pieces of most of that stay. I have no memory of any doctors during that time, in-fact, in my follow-up visits with the doctors that had seen me in the hospital, I thought I was meeting them for the first time. Due to the bleed in my brain, I was left with a visual field cut on the left side of both eyes, but no physical defects of any kind. In the hospital I was totally unaware of what I couldn’t see, and it was only after I returned home that I realized how bad it really was. When I was being driven home it was a nightmare for me, as my eyes could not take in everything around me as I looked out the window. I had to cover my eyes and cried all the way home.

***“In the hospital I was totally unaware of what I couldn’t see, and it was only after I returned home that I realized how bad it really was.”***

Back in the house, my only comfort was my dogs and my bed, where I could close my eyes and sleep. It was impossible to read or watch television, and the computer was my enemy. Any artificial lighting was a problem, especially fluorescent lighting, which would disorient me and make me feel sick. My only comfort with lighting was the natural light of day, although if too sunny, the light was too intense for me. On overcast days it was worse, because it was very hard to see and everything was even dimmer for me and made me more depressed. I mention all these things, because it caused me much anxiety and panic, which caused yet another problem for me. I experienced all these post stroke side effects, because of my visual field deficit. I experienced them alone, because no one gave me a heads up on how it might be.

Going back to the hospital and upon being discharged, if someone did have a conversation with me, I don’t remember it. What I do remember is a therapist wanting to see if I could dress myself and brush my teeth, which I could. I remember receiving a lot of paperwork and signing a number of things, which I couldn’t tell you what they were and I couldn’t read them anyway. The only documents I do remember getting were the prescriptions that I was told to fill. There were so many of them I asked why and which ones I really needed. In the hospital, I was only taking my small dose of Synthroid, but now I had an anti-seizure medication (which I was not even aware I was taking in the hospital), and a pain medication. Other than that, it was just my vitamin supplements. The prescription list included 3 different types of pain medication and again, I asked why. I was told they were different strengths and I might need them. To me this was a problem, because if I wasn’t proactive enough to ask, I might have filled them all and taken them all and, undoubtedly put me at risk for overdose. I never did fill all the pain medications, just the one I was last taking in the hospital.

Physical therapy appointments were made for me right away and actually, the very next day after being discharged. Although I am appreciative for being able to receive this therapy, I was too emotional to start therapy one day after being home. Also, I couldn’t drive, so if I didn’t have family and friends that came to my aid and some from other states to take care of me, how would I have gotten there? After a few weeks had gone by and most of my care-givers had left, I did ask this question and was told transportation was not provided. Back to the therapy itself, what I remember from therapy was that it was more about my memory and how I processed information rather than my visual field deficit. All were important for sure, but very little information was given to me about my visual field deficit and problems it would cause.

***“Having a medical person come to the house within a few days or a week of being home, would have made all the difference in the world for me… Knowing what to expect, what was normal and what was not, would have reduced my anxiety and ultimately improved my recovery.”***

In summary, it would have been extremely beneficial to me and most everyone, if there was a more streamlined transition from hospital to home. Having a medical person come to the house within a few days or a week of being home, would have made all the difference in the world for me and my caregiver. I wouldn’t have been so terrified and we both could have asked the questions we needed to ask, eliminating numerous calls to the doctors and nurses. For me, all phone calls were responded to in a timely manner, but it would have been such a comfort for a medical person to do periodic home visits. Knowing what to expect, what was normal and what was not, would have reduced my anxiety and ultimately improved my recovery.

**62 year old, white male, living in urban NC, physician and full-time professor with nearly 30 years of medical experience**

It was almost Christmas time 2012. I was looking forward to time spent at our church and then a week of peaceful time up in the mountains. Suddenly, I was awakened by a terrible headache. I had been getting a series of them every month, but this one was just too terrible to stay at home. In the emergency room they looked me over. Thankfully they felt my physical exam was completely normal. They gave me something for the pain, but still were concerned. They ordered a CAT scan which turned out to be completely normal. By that time the pain was getting better. After watching me some more they agreed to send me home. I went home and got a few hours of sleep. I arose and got ready for the nursing home. While there on rounds I became unconscious. Luckily, I was in a good place surrounded by clinicians. They wheeled me off to the hospital where this time, both my carotid (neck) vessels were occluded. At that point they tried to place bypasses on each side, but it did not work. Finally they did get some flow on the right side and placed a stent. Unfortunately, the left side was still blocked and there was associated left anterior vessel bleeding. This left me with a dense right-sided hemiplegia, great difficulty with speech and poor alertness. (This part of the story was put together by many other people. My memories of these details simply do not exist.)

***“Preparing the arrangements at home was left to the family. There was no home visit, and no directions were given to help with getting other services.”***

My loving wife, Joanie, was the caretaker from Day 1. As an eighth grade school teacher, she asked all the necessary questions. For the first two weeks, during her Christmas vacation, my wife stayed long hours learning the ropes. Then it was time to return to teaching. She knew she could work while I was under good care in the Sticht Center. The question was, “What was to happen when I came home?” The therapists prepared for my return home by training my wife and ordering the necessary equipment (wheelchair, shower seat, cane). Discharge medication instruction and prescriptions were given. Appointments were made with Comp Rehab for speech and physical therapy. Preparing the arrangements at home was left to the family. There was no home visit, and no directions were given to help with getting other services. My wife, on her own, arranged to hire friends to sit with me while she continued to teach. I feel that this could have been handled more effectively.

Over the next two months, I continued improving in speech and physical therapy. The physical therapy services were excellent, challenging me to gain strength and improve my mobility. The speech therapy was really not very helpful; most appointments were focused on assessment with very little direction to develop strategies for restarting my language capability. In response to this, my wife researched nationally known centers for aphasia and arranged for me to attend the program at the University of Michigan for the entire month of April. She and other family members stayed with me in Michigan while I attended the excellent, but exhausting, program. During the month of March, I began attending sessions at the North Carolina Center for Cognitive Rehabilitation. My wife found out about this program when its director came to offer the service at the Sticht Center. Fourteen months later, I still attend the Center and will volunteer there next fall.

***“I got what I needed (the basics), but not the superb without my wife pushing the system and spending lots of money.”***

When we first noticed movement in the right hand at 2 months, we demanded that the Occupational therapists prescribe an electrical nerve stimulating device that helped me develop more movement in my arm and hand. After several months, I again had to press for a SAEBO (special hand) device that I used over the summer to develop functional hand movements.

All in all, I got what I needed (the basics), but not the superb without my wife pushing the system and spending lots of money.
