# Supplementary material for: How engagement of a diverse set of stakeholders shaped the design, implementation, and dissemination of a multicenter pragmatic trial of stroke transitional care: The COMPASS study
Source: J Clin Transl Sci. 2020 Nov 5;5(1):e60. doi: 10.1017/cts.2020.552 (PMC8057438; doi:10.1017/cts.2020.552)
Supplement: Supplementary file 1 [file S205986612000552Xsup.zip › S205986612000552Xsup004.docx]

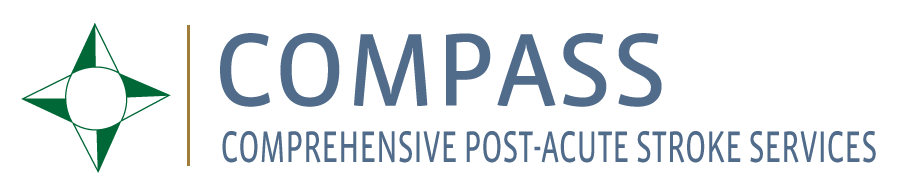


1 November 2015

Dear Members of the Sanford/Lee County Stroke Support Group:

Thank you for your thoughtful comments on what patients and their families need during recovery after a stroke. We have incorporated your ideas into the COMPASS study. In particular, your ideas have shaped the way we will train the doctors, nurses, pharmacists and therapists who deliver the COMPASS intervention to stroke patients across North Carolina next year. Thank you for partnering with us on this important project.

**How the COMPASS Study Has Used Your Input**

1. **Based on your feedback we will make sure that patients and their family members get the information you said they need to have:**

- We will provide information to patients and their families early after a stroke on how to manage their health and how to prevent another stroke (manage their blood pressure, be active, take medications consistently, etc). We will tell them this every time we interact with them in person or over the phone during the study.
- When speaking with patients and their families, we will emphasize the need for rehab -- even if they don’t want to hear it, and answer their questions to make sure they understand how important therapy is to their best recovery. In this way, they will have a plan for recovery and start rehab right away.
- We will emphasize that recovery from stroke is a process that can take many years, and that “there is no cap on recovery.”

1. **You told us that stroke survivors can find follow-up calls difficult to follow. Based on your feedback:**
   - We will train the Post-Acute Care Coordinators to involve family caregivers starting with the first follow-up call 2 days after discharge.
2. **You told us that clinicians need to stay aware of the fact that stroke survivors are real people, with feelings and with complex lives now in upheaval. Based on your feedback we will:**
   - Train all healthcare providers involved in the intervention on what it means to give Patient-Centered Care.
   - We will ask stroke survivors and their family caregivers to review the training materials to make sure we capture what matters to patients.
3. **You made several comments for how we can help patients manage their medications at home. We reviewed these ideas with the pharmacist and the stroke neurologist who have developed a new Medication Management Toolkit that patients and their primary care providers will use in the study. In a user-friendly way, we will:**

- Give patients a list of their medicines
- Explain why they are taking their medicines
- Help them take their medicine’s consistently (like using pill boxes, setting alarms to remind them)
- Give doctors a list of the most affordable medicines for each type of insurance
- We will also train healthcare providers in the study on how to help patients manage their medications.

1. **You voiced concern with caring for all patients under the same protocol.** **Based on this feedback:**

- The COMPASS study will create individualized care plans to guide each stroke survivor’s recovery.
- COMPASS will also train primary care doctors and other providers to appreciate the fact that “not all strokes are exactly the same – in fact you could have the same size stroke in two different people and one person could be devastated and the other recover completely.”

1. **You voiced concern that stroke survivors have challenges accessing services. Based on your feedback:**

- Therapists involved in the study will be given training and reference documents to help meet the needs of patients with all types of health insurance.
- Therapists involved in the study will be trained to take advantage of community resources that survivors can use to improve recovery.
- The COMPASS website will have information and resources that can help survivors improve their recovery at home (for example, speech therapy programs they can do at home, physical activity programs in their community)
- For each hospital in the study, we are mapping out relevant community resources that can support recovery. Information specific to the survivor and caregiver’s needs will be given to patients during the follow up visit 7-14 day after discharge, so that they know where to find the services they need.

Again, thank you for partnering with us on this important project.

Please feel free to reach out to me if you have any questions or comments:

Sabina Gesell, Ph.D.

Wake Forest School of Medicine
Tel: 336-713-8738

[sgesell@wakehealth.edu](mailto:sgesell@wakehealth.edu)

Please visit our study website: <https://www.nccompass-study.org/>
