# Supplementary material for: How engagement of a diverse set of stakeholders shaped the design, implementation, and dissemination of a multicenter pragmatic trial of stroke transitional care: The COMPASS study
Source: J Clin Transl Sci. 2020 Nov 5;5(1):e60. doi: 10.1017/cts.2020.552 (PMC8057438; doi:10.1017/cts.2020.552)
Supplement: Supplementary file 1 [file S205986612000552Xsup.zip › S205986612000552Xsup001.doc]

**COMPASS Stakeholder Reimbursement Policy and Procedures**

Original Effective Date: Oct 1, 2015

Last Revised Date: Oct 6, 2020

**Purpose**

As described in the COMPASS study grant and protocol, one primary goal of PCORI and the COMPASS study is to create implementable and pragmatic programs that meet the immediate needs of stakeholders. As such, it will be critical to gather feedback, guidance and support from stakeholders in all phases of the study including planning, implementation, sustainability and dissemination.

The purpose of COMPASS Stakeholder Reimbursement Policy and Procedures is to establish guidance for study investigators on engaging stakeholders for COMPASS related activities. Specifically, this policy will outline the type of stakeholders that will receive reimbursements to ensure consistency across the study, and adherence to PCORI principals of reciprocal relationships, trust and transparency [1].

A secondary purpose is to ensure that processes for reimbursement are outlined to ensure stakeholders are reimbursed within a reasonable time frame, that reimbursements are well documented for audits and within our allotted budget.

This policy seeks to align with the guidance put forth by PCORI [1] while meeting the conservative budget allotted for COMPASS stakeholder activities.

**Stakeholder and Study Personnel Definitions**

*Investigators*: COMPASS Study personnel receiving effort to participate in COMPASS study activities.

*Stakeholder-Investigators*: COMPASS Study personnel that are both stakeholders and study investigators. This type of stakeholder receives effort to participate in COMPASS study activities.

*Stakeholders:* Any COMPASS Study stakeholder who will be impacted by the roll-out of the COMPASS Study. There are several categories of stakeholders:

*Patient and Caregiver Stakeholders:* Stakeholders that will not receive financial and/or professional benefit from participating in the implementation of the COMPASS study.

This includes: Patients and Caregivers

*Health Professional Stakeholders:* Stakeholders that may receive financial and/or professional benefit from the implementation of the COMPASS Study.

This includes: Health professionals - Home Health, Outpatient Therapists, Pharmacists, Primary Care Physicians, Stroke Coordinators, etc.

*Corporate Stakeholders:* Stakeholders that will receive financial and/or professional gain from the implementation of the COMPASS Study.

This includes: American Heart Association/American Stroke Association, Elsevier, etc.

**Policy**

1. All stakeholder activities and stakeholder travel that will require reimbursement from the COMPASS Study require approval by financial manager (or project manager) **before** activities take place. For approval, you can email or use the ***COMPASS Engagement Approval Form*** to identify purpose of the activity, estimate expense and gain approval.
2. There are two types of engagement activities: time participating in study-related meetings (defined as non-research activities by the IRB) and structured focus groups or interviews (defined as research by the IRB).
   a. **Reimbursement for Time in Meetings:** *Patient and Caregiver Stakeholders* will be eligible for compensation for time spent on the COMPASS Study. This will be at the rate of $20.00/hour.
      i. *Investigators*, *Stakeholder-investigators*, *Health Professional Stakeholders* and *Corporate Stakeholders* will receive other benefits (financial and/or professional benefits) for time spent on the COMPASS Study and will therefore not be reimbursed for time spent in meetings.
   b. **Reimbursement for Focus Group or Interviews Participation:** *Patient and Caregiver Stakeholders* and *Health Professional Stakeholders* will be compensated for time spent in focus groups at the rate of $25.00/hour.
      i. *Investigators*, *Stakeholder-investigators* and *Corporate Stakeholders* will not be reimbursed for focus group participation as they will receive other benefits (financial and/or professional benefits) for focus group participation.
3. **Reimbursement for Travel**: Stakeholders will rarely be asked to travel to non-regional meetings. Every effort will be made to engage stakeholders remotely. In the case that a stakeholder is asked to travel to a non-regional meeting they will be reimbursed for their travel at the government mileage rate currently $0.575/mile [2].
   i. **Note:** *Patient and Caregiver Stakeholders*, *Health Professional Stakeholders*, *Investigators*, *Stakeholder-investigators* are eligible for reimbursement for travel related to engagement activities. *Corporate Stakeholders* will not be reimbursed for travel.
4. Stakeholders will need information on what they are being asked to contribute and how they will be compensated for their valuable time and expertise. To ensure that stakeholders have this information, please notify stakeholders in writing on the activity and compensation before the event.
5. In order to monitor the limited budget for stakeholder engagement activities, we ask that all engagement activities receive pre-approval. It is highly unlikely that we will be able to reimburse stakeholders for activities that were not pre-approved.
6. When a COMPASS engagement activity is changed or cancelled the study team will do their best to quickly communicate this information to any stakeholders involved. Below is guidance for study investigators and important considerations for stakeholders:
   a. COMPASS Study team members will make every effort to keep meetings on schedule.
   b. In the case that a meeting is longer than expected (i.e. a 1 hour meeting becomes 3 hours), stakeholders should use her/his judgement on if they are willing to continue to stay in the meeting as additional time spent in meetings that are running over may not be reimbursed.
   c. In the unfortunate event that a meeting is cancelled, it will not be possible to reimburse stakeholders for planned activities that were cancelled. If a cancellation is required, the study team will try to cancel with as much advanced notice as possible.
7. Oversight and guidance will be provided by the Engagement Committee and the Executive Leadership Committee (which includes PCORI members) to best determine how to use the limited funds dedicated to stakeholder engagement.
8. Stakeholders that are supporting COMPASS on a reoccurring basis are encouraged to accept compensation for their time and expertise.
   a. Patients can decline payment if they would like to do so, however:
      i. In the case that the stakeholder refuses compensation, this should be documented in writing for tracking purposes.
      ii. Always offer compensation for all study activities even to stakeholders who have declined compensation in the past.
      iii. The engagement committee should regularly assess stakeholder contributions and level of compensation and check in with stakeholders who are not accepting payment to ensure there is equity in compensation.

**Procedure for Reimbursement of Stakeholder Engagement**

Before the activity

1. Gain approval before the engagement activity to ensure there is funds available for reimbursement.
2. If necessary, have the stakeholder fill out a W9 Form, return the form to Project Manager so the administrative team can establish the stakeholder as a vendor.

After the activity

1. Request payment from the project manager after the event. Include the description of payment, confirm the amount of time the stakeholder spent on the activity. Be sure to log the stakeholder activity in the RedCap Engagement Tracker.

**Approvals and Contact Information:**

Stakeholder reimbursements must be approved by [Insert name/email]

**Attachments:**

1. COMPASS Engagement Approval Form (CEAF)

**Reference:**

1. PCORI. Compensation Framework: <http://www.pcori.org/sites/default/files/PCORI-Compensation-Framework-for-Engaged-Research-Partners.pdf> [last access: Oct2020]
2. <http://www.gsa.gov/portal/content/100715> [last access: Oct2020]
